# The prognostic value of the nodal ratio in N1 breast cancer

**DOI:** 10.1186/1748-717X-6-131

**Published:** 2011-10-06

**Authors:** Tae Jin Han, Eun Young Kang, Wan Jeon, Sung-Won Kim, Jee Hyun Kim, Yu Jung Kim, So Yeon Park, Jae Sung Kim, In Ah Kim

**Affiliations:** 1 Department of Radiation Oncology, Seoul National University, Bundang Hospital, 166 Gumiro Seongnamsi Kyeonggido, 463-707, Korea; 2 Breast Care Center, Seoul National University, Bundang Hospital, Korea

**Keywords:** breast cancer, N1, nodal ratio, prognostic factor

## Abstract

**Background:**

Although the nodal ratio (NR) has been recognized as a prognostic factor in breast cancer, its clinical implication in patients with 1-3 positive nodes (N1) remains unclear. Here, we evaluated the prognostic value of the NR and identified other clinico-pathologic variables associated with poor prognosis in these patients.

**Methods:**

We analyzed 130 patients with N1 invasive breast cancer who were treated at Seoul National University Bundang Hospital from March 2003 to December 2007. Disease-free survival (DFS), locoregional recurrence-free survival (LRRFS), and distant metastasis-free survival (DMFS) were compared according to the NR with a cut-off value of 0.15.

**Results:**

We followed patients' recovery for a median duration of 59 months. An NR > 0.15 was found in 23.1% of patients, and a median of 18 nodes were dissected per patient (range 1-59). The NR was statistically independent from other prognostic variables, such as patient age, T stage, extent of surgery, pathologic factors in the chi square test. On univariate analysis, patients with a NR > 0.15 had significantly lower 5-year LRRFS (88.7% vs. 97.9%, p = 0.033) and 5-year DMFS (81.3% vs. 96.4%, p = 0.029) and marginally lower 5-year DFS (81.3% vs. 94.0%, p = 0.069) than those with a NR ≤0.15, respectively. Since the predictive power of the NR was found to differ with diverse clinical and pathologic variables, we performed adjusted analysis stratified by age, pathologic characteristics, and adjuvant treatments. Only young patients with a NR > 0.15 showed significantly lower DFS (p = 0.027) as well as those presenting an unfavorable pathologic profile such as advanced T stage (p = 0.034), histologic grade 3 (p = 0.034), positive lymphovascular invasion (p = 0.037), involved resection margin (p = 0.007), and no chemotherapy (p = 0.014) or regional radiotherapy treatment (p = 0.039). On multivariate analysis, a NR > 0.15 was significantly associated with lower DFS (p = 0.043) and DMFS (p = 0.012), but not LRRFS (p = 0.064).

**Conclusions:**

A NR > 0.15 was associated with an increased risk of recurrence, especially in young patients with unfavorable pathologic profiles.

## Background

The presence of axillary lymph node metastasis is one of the most important factors affecting prognosis in patients with breast cancer [1]. According to the current 7th edition of the American Joint Committee on Cancer staging system, N stage in breast cancer is solely determined by the number of positive nodes [2]. In patients with inappropriately dissected axillary nodes, however, a discrepancy may exist between the absolute number of positive nodes and the substantive extent of axillary node metastasis [3]. Therefore, the nodal ratio (NR), defined as the absolute number of involved nodes/number of excised nodes, has been suggested to address this discrepancy [4]. Recent studies have shown the prognostic value of the NR and even proposed the possibility of NR as an alternative or a complement to N staging in node-positive breast cancer [5-13]. However, no consensus has been reached for the appropriate criteria to discriminate between low- and high-risk groups of NR for breast cancer with 1-3 positive nodes.

In the current study, we evaluated the prognostic value of the NR and identified other clinico-pathologic variables associated with poor prognosis in N1 breast cancer patients.

## Methods

### Patients

We retrospectively analyzed 130 patients with N1 invasive breast cancer who were treated at Seoul National University Bundang Hospital (SNUBH) from March 2003 to December 2007. Patients who had received neoadjuvant chemotherapy prior to surgery were excluded. We collected not only treatment modality information such as type of surgery, type of systemic treatment, and radiation field, but also detailed clinico-pathologic prognostic factors such as age, pathologic stage, histologic type and grade, number of excised and positive nodes, estrogen/progesterone receptor (ER/PR) status, human epithelial growth factor receptor family 2 (HER2) status, presence of extracapsular extension (ECE), presence of lymphovascular invasion (LVI), and resection margin status. A close margin was defined as the presence of invasive carcinoma within 2 mm of the surgical margin of resection.

### Patient grouping according to the nodal ratio

We categorized the patients into two NR groups: low NR (LNR; ≤0.15) and high NR (HNR; > 0.15). Disease-free survival (DFS), locoregional recurrence-free survival (LRRFS), and distant metastasis-free survival (DMFS) were compared between groups. We defined locoregional recurrence as the first site of recurrence involving residual breast or chest wall (local) tissue and/or axillary, supra- or infraclavicular, and internal mammary nodes (regional). For cases in which locoregional recurrence and distant metastasis simultaneously occurred, we counted both failure patterns.

### Statistical analysis

To make comparisons between the two groups, we used the chi-square test or Fisher's exact test for categorical data and independent sample t-test for continuous data. The Kaplan-Meier method was used for DFS, LRRFS, and DMFS probability, and survival according to different variables was compared by the log-rank test. The Cox proportional hazard method was used to perform multivariate analysis for predictors of survival. We included variables that showed significance in the univariate analysis or were otherwise were considered to be confounders in the multivariate analysis. All statistical analyses were performed with Statistical Package for the Social Sciences (version 17.0; SPSS, Chicago, IL). We considered *p *values equal to or less than 0.05 to be statistically significant.

## Results

### Patient and tumor characteristics

Of the 130 patients, the LNR group included 100 patients and the HNR group included 30 patients. Patient characteristics for these two groups are summarized in Table 1. The median number of excised nodes per patient was 18 (range, 1-59) for both groups combined, and was significantly higher in the LNR group than in the HNR group (20 vs. 7, p < 0.001). RT was used to treat 46 (46%) LNR patients and 20 (66.7%) HNR patients; among these, regional RT was more frequently used in the HNR group (50.0% vs. 10.9%, p = 0.001). The local RT field consisted of the whole breast or chest wall only. In contrast, supraclavicular lymph nodes and/or internal mammary lymph nodes were included in the locoregional RT field. Chemotherapy was used to treat 92 (92%) LNR patients and 25 (83.3%) HNR patients. Taxane-containing regimens such as AC (adriamycin and cyclophosphamide) were most frequently prescribed. The tumor characteristics in the two groups are summarized in Table 2. Infiltrating ductal carcinoma was the most frequent tumor histology in both groups, but was more dominant in the LNR group (92.0% vs. 73.3%, p = 0.011). The NR was a statistically independent variable from other prognostic variables including patient age, extent of surgery, and pathologic factors such as ECE, LVI, tumor grade, margin status, ER/PR, and HER2 status.

**Table 1:** Patient characteristics

	No. of patients (%)			
			
	NR < 0.15 (n = 100)	NR > 0.15 (n = 30)	p value	Total (n = 130)
Age (years)			0.225	
median (range)	46 (25-79)	50 (32-82)		47 (25-82)
Excised LN (No.)			< 0.001	
median (range)	20 (7-59)	7 (1-18)		18 (1-59)
Breast resection			0.290	
BCS	49 (49.0)	18 (60.0)		67 (51.5)
MRM	51 (51.0)	12 (40.0)		63 (48.5)
LN resection			0.071	
SLNB only	3 (3.0)	4 (13.3)		7 (5.4)
SLNB + ALND	68 (68.0)	20 (66.7)		88 (67.7)
ALND	29 (29.0)	6 (20.0)		35 (26.9)
Radiotherapy			0.047	
no	54 (54.0)	10 (33.3)		64 (49.2)
yes	46 (46.0)	20 (66.7)		66 (50.8)
Extent of radiotherapy			0.001	
local	41 (89.1)	10 (50.0)		51 (77.2)
locoregional	5 (10.9)	10 (50.0)		15 (22.8)
Chemotherapy			0.176	
no	8 (8.0)	5 (16.7)		13 (10.0)
yes	92 (92.0)	25 (83.3)		117 (90.0)
Regimen			0.141	
CMF	12 (13.0)	7 (28.0)		19 (16.2)
FEC/FAC	31 (33.7)	9 (36.0)		40 (34.2)
ACT	49 (53.3)	9 (36.0)		58 (49.6)

*Abbreviations*: LN, lymph node; BCS, breast-conserving surgery; MRM, modified radical mastectomy; SLNBx, sentinel lymph node biopsy; ALND, axillary lymph node dissection; CMF, cyclophosphamide/methotrexate/5-fluorouracil; FEC, 5-FU/epirubicin/cyclophosphamide; FAC, 5-FU/adriamycin/cyclophosphamide; ACT, adriamycin/cyclophosphamide/paclitaxel

**Table 2:** Tumor characteristics

	No. of patients (%)			
			
	NR ≤0.15 (n = 100)	NR > 0.15 (n = 30)	p value	Total (n = 130)
Histology			0.011	
IDC	92 (92.0)	22 (73.3)		114 (87.7)
others	8 (8.0)	8 (26.7)		16 (12.3)
T stage			0.166	
T1a/T1b	8 (8.0)	6 (20.0)		14 (10.8)
T1c	42 (42.0)	12 (40.0)		54 (41.5)
T2	49 (49.0)	10 (33.3)		59 (45.4)
T3	1 (1.0)	2 (6.7)		3 (2.3)
ER			0.459	
(-)	23 (23.0)	5 (16.7)		28 (21.5)
(+)	77 (77.0)	25 (83.3)		102 (78.5)
PR			0.155	
(-)	41 (41.0)	8 (26.7)		49 (37.7)
(+)	59 (59.0)	22 (73.3)		81 (62.3)
HER2			0.866	
(-)	72 (72.0)	25 (83.3)		107 (82.3)
(+)	18 (18.0)	5 (16.7)		23 (17.7)
unknown	10 (10.0)	0 (0.0)		
ECE			0.183	
(-)	74 (74.0)	22 (73.4)		97 (74.6)
(+)	26 (26.0)	7 (23.3)		33 (25.4)
unknown	0 (0.0)	1 (3.3)		
LVI			0.175	
(-)	52 (52.0)	14 (47.7)		67 (51.5)
(+)	48 (48.0)	15 (50.0)		63 (48.5)
unknown	0 (0.0)	1 (3.3)		
Resection margin			0.868	
(-)	91 (91.0)	27 (90.0)		118 (90.8)
close or (+)	9 (9.0)	3 (10.0)		12 (9.2)
Tumor grade			0.297	
G1/G2	63 (63.0)	22 (73.3)		85 (65.4)
G3	37 (37.0)	8 (26.7)		45 (34.6)

*Abbreviations*: NR, nodal ratio; IDC, infiltrating ductal carcinoma; ER, estrogen receptor; PR, progesterone receptor; ECE, extracapsular extension; LVI, lymphovascular invasion; RM, resection margin

### Follow-up and patterns of failure

We followed patients' recovery for a median duration of 59 months (range, 10-89 months) for both groups although the LNR group had a longer duration of follow-up (p = 0.013). Both groups showed distant metastasis as the dominant failure pattern in eight of nine patients who experienced any failures. These details are summarized in Table 3.

**Table 3:** Clinical status and patterns of failure

	No. of patients (%)		
		
	NR ≤0.15 (n = 100)	NR> 0.15 (n = 30)	Total (n = 130)
Follow-up (months)			
median (range)	61 (10-89)	48 (26-78)	59 (10-89)
Clinical Status			
NED	96 (96.0)	26 (86.6)	122 (93.9)
alive with disease	4 (4.0)	2 (6.7)	6 (4.6)
cause-specific death	0 (0.0)	2 (6.7)	2 (1.5)
intercurrent death	0 (0.0)	0 (0.0)	0 (0.0)
Patterns of failure			
LRR only	1 (20.0)	0 (0.0)	1 (11.0)
DM only	3 (60.0)	1 (25.0)	4 (44.5)
LRR+DM	1 (20.0)	3 (75.0)	4 (44.5)

*Abbreviations*: NR, nodal ratio; NED, no evidence of disease; LRR, locoregional recurrence; DM, distant metastasis

### Univariate analysis of different prognostic factors

**Figure 1:**
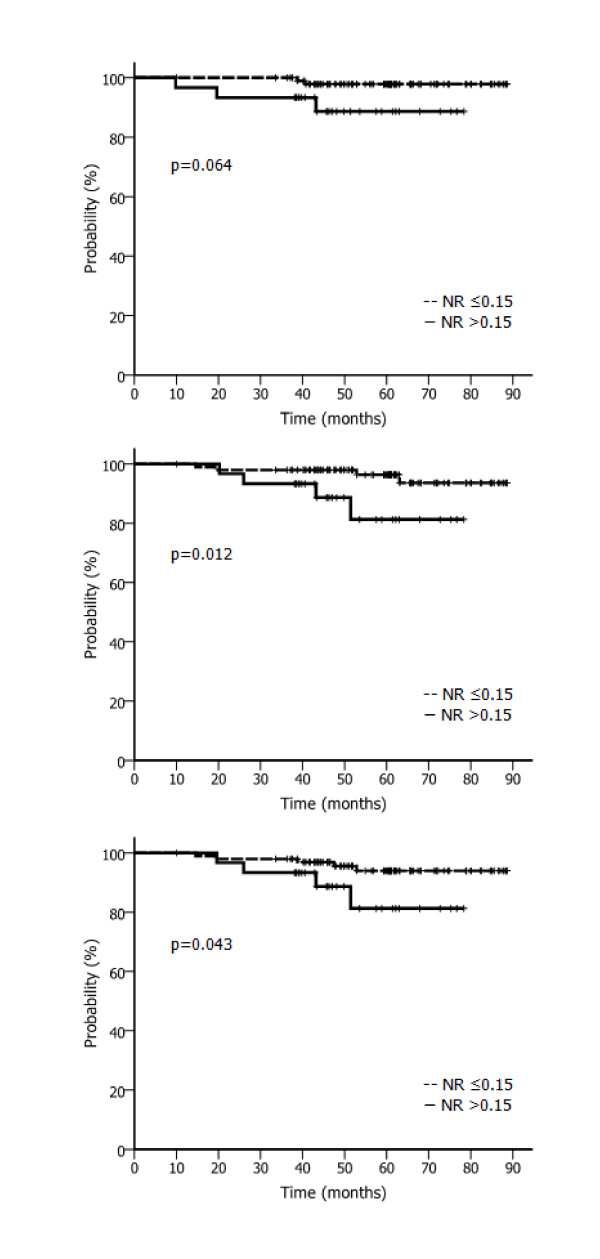
**LRRFS, DMFS, and DFS according to NR**. (a) LRRFS according to NR. (b) DMFS according to NR. (c) DFS according to NR. Abbreviations: LRRFS, locoregional recurrence-free survival; DMFS, distant metastasis-free survival; DFS, disease-free survival; NR, nodal ratio.

The univariate analysis results for prognostic factors are summarized in Table 4. According to the univariate analysis, patients with a NR > 0.15 had significantly lower 5-year LRRFS (88.7% vs. 97.9%, p = 0.033) and 5-year DMFS (81.3% vs. 96.4%, p = 0.029) and marginally lower 5-year DFS (81.3% vs. 94.0%, p = 0.069) than those with a NR ≤0.15 (Figure 1).

**Table 4:** Univariate analysis for 5-year LRRFS, DMFS, and DFS

Variable	No. of pts	5-year LRRFS (%)	5-year DMFS (%)	5-year DFS (%)
Age				
≤40	31	92.7	84.3	84.3
> 40	99	96.8	96.2	93.7
		p = 0.348	p = 0.078	p = 0.138
T stage				
T1	68	96.5	96.7	94.6
T2/3	62	95.0	89.5	87.7
		p = 0.578	p = 0.108	p = 0.229
Nodal ratio				
≤0.15	100	97.9	96.4	94.0
> 0.15	30	88.7	81.3	81.3
		p = 0.033	p = 0.029	p = 0.069
ER				
(-)	28	96.4	96.4	95.4
(+)	102	95.6	92.3	89.8
		p = 0.895	p = 0.462	p = 0.374
PR				
(-)	49	98.0	98.0	98.0
(+)	81	94.5	90.5	87.5
		p = 0.403	p = 0.111	p = 0.087
HER2				
(-) or unknown	107	94.4	92.0	89.7
(+)	23	100.0	100.0	100.0
		p = 0.421	p = 0.180	p = 0.163
Histologic grade				
G1/G2	85	97.3	94.3	92.7
G3	45	93.1	91.7	89.2
		p = 0.203	p = 0.354	p = 0.510
ECE				
(-) or unknown	97	95.5	91.3	90.2
(+)	33	96.8	100.0	95.2
		p = 0.639	p = 0.117	p = 0.332
LVI				
(-) or unknown	67	96.4	95.0	93.3
(+)	63	95.1	91.6	89.7
		p = 0.639	p = 0.927	p = 0.768
RM				
(-)	118	96.2	94.9	92.7
close or (+)	12	91.7	83.3	83.3
		p = 0.431	p = 0.213	p = 0.268
Surgery				
BCS	67	98.5	95.5	95.5
MRM	63	93.1	91.9	88.1
		p = 0.160	p = 0.556	p = 0.345
Regional RT				
no	115	96.2	93.3	91.2
yes	15	93.3	93.3	93.3
		p = 0.503	p = 0.976	p = 0.935
Chemotherapy				
no	13	92.3	79.1	79.1
yes	117	96.3	94.9	92.8
		p = 0.401	p = 0.104	p = 0.173

*Abbreviations*: LRRFS, locoregional recurrence-free survival; DMFS, distant metastasis-free survival; DFS, disease-free survival; ER, estrogen receptor; PR, progesterone receptor; ECE, extracapsular extension; LVI, lymphovascular invasion; RM, resection margin; BCS, breast-conserving surgery; MRM, modified radical mastectomy

### The effect of NR on DFS stratified by other prognostic factors

**Figure 2:**
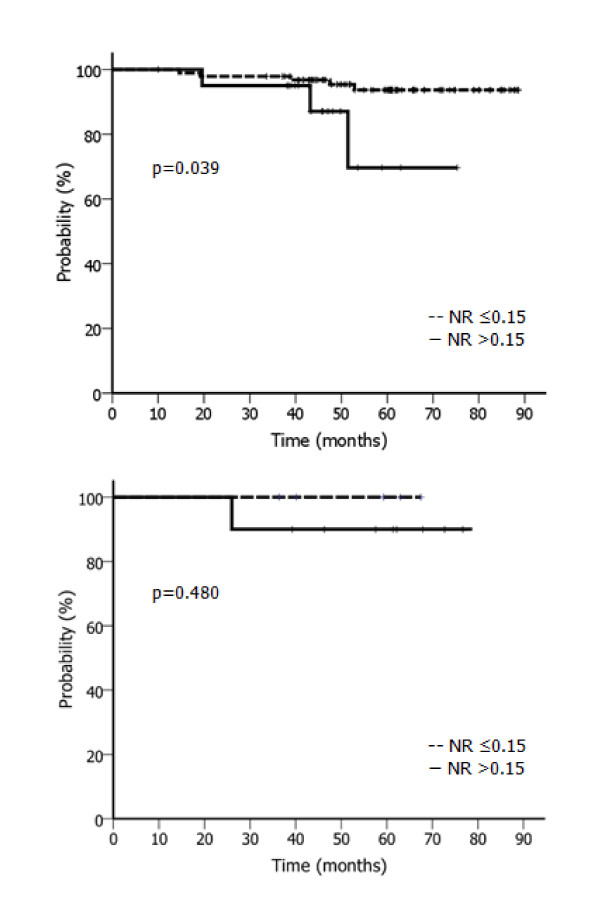
**Adjusted analysis for DFS with or without regional RT**. (a) DFS according to nodal ratio with regional RT. (b) DFS according to nodal ratio without regional RT. Abbreviations: DFS, disease-free survival; NR, nodal ratio.

Since the prognostic power of the NR was found to differ according to diverse clinical and pathologic variables, we performed adjusted analysis stratified by age, pathologic characteristics, and adjuvant treatments. The HNR group showed significantly lower 5-year DFS exclusively in those presenting an unfavorable clinico-pathologic profile: young age (p = 0.027), advanced T stage (p = 0.034), high grade (p = 0.034), the presence of LVI (p = 0.037), involved resection margin (p = 0.007) and the lack of chemotherapy (p = 0.014) or regional RT (p = 0.039; Table 5). The DFS curves according to NR with and without regional RT are presented in Figure 2.

**Table 5:** Adjusted analysis for DFS

		5-year DFS (%)		
			
	No. of pts	NR ≤0.15	NR > 0.15	p value
Age				
≤40	31	90.4	53.3	0.027
> 40	99	95.4	87.3	0.361
T stage				
T1	68	95.4	90.9	0.586
T2/3	62	92.3	66.7	0.034
Histologic grade				
G1/2	85	94.7	84.7	0.387
G3	45	92.6	75.0	0.034
LVI				
(-) or unknown	67	94.0	87.5	0.849
(+)	63	93.8	75.8	0.037
RM				
(-)	118	93.2	90.9	0.594
close or (+)	12	100.0	33.3	0.007
Regional RT				
no	115	93.7	69.7	0.039
yes	15	100.0	90.0	0.480
Chemotherapy				
no	13	100.0	80.0	0.014
yes	117	93.4	90.7	0.556

*Abbreviations*: DFS, disease-free survival; NR, nodal ratio; ECE, extracapsular extension; LVI, lymphovascular invasion; RM, resection margin

### Multivariate analysis of different prognostic factors

According to the multivariate analysis, a NR > 0.15 was significantly associated with lower DFS (p = 0.043) and DMFS (p = 0.012) but not LRRFS (p = 0.064; Table 6). Patients not treated with chemotherapy showed a tendency of increased distant metastasis.

**Table 6:** Multivariate analysis for LRRFS, DMFS, and DFS

	Hazard ratio (95% CI)		
	
Variables	Locoregional recurrence	Distant metastasis	Any failure
Age			
(> 40 vs. ≤40)	0.62 (0.07-5.27)	0.07 (0.01-0.80)	0.22 (0.04-1.36)
	p = 0.664	p = 0.033	p = 0.104
T stage			
(2-3 vs. 1)	1.02 (0.11-9.02)	6.09 (0.83-44.72)	2.58 (0.52-12.82)
	p = 0.989	p = 0.076	p = 0.247
NR			
(> 0.15 vs. ≤0.15)	7.38 (0.89-61.25)	11.22 (1.70-74.04)	5.06 (1.05-24.37)
	p = 0.064	p = 0.012	p = 0.043
Histologic grade			
(3 vs. 1-2)	5.86 (0.48-71.23)	1.26 (0.22-7.33)	1.33 (0.28-6.30)
	p = 0.166	p = 0.795	p = 0.724
LVI			
(positive vs. negative or unknown)	0.90 (0.10-8.14)	0.38 (0.06-2.32)	0.71 (0.15-3.25)
	p = 0.927	p = 0.294	p = 0.654
RM			
(close or positive vs. negative)	1.36 (0.08-22.32)	1.87 (0.22-16.26)	1.62 (0.23-11.30)
	p = 0.829	p = 0.569	p = 0.624
Surgery			
(MRM vs. BCS)	4.62 (0.37-57.31)	0.57 (0.87-3.78)	1.21 (0.24-6.22)
	p = 0.234	p = 0.563	p = 0.819
Regional RT			
(yes vs. no)	1.70 (0.12-24.18)	0.64 (0.06-6.95)	0.59 (0.06-5.92)
	p = 0.695	p = 0.717	p = 0.653
Chemotherapy			
(yes vs. no)	0.17 (0.01-5.22)	0.15 (0.01-1.68)	0.27 (0.03-2.41)
	p = 0.311	p = 0.125	p = 0.243

*Abbreviations*: CI, confidence interval; LRRFS, locoregional recurrence-free survival; DMFS, distant metastasis-free survival; DFS, disease-free survival; LVI, lymphovascular invasion; RM, resection margin; BCS, breast-conserving surgery; MRM, modified radical mastectomy

## Discussion

The prognostic value of NR is supported by several studies [4-13]. Vinh-Hung et al. reported the superiority of NR over pN stage in predicting disease-specific survival, and Danko et al. revealed that the prognostic value of NR for disease-free survival remained significant even when stratified by pN stage [8,10]. Recently, Ahn et al. showed that NR is a better predictor of disease-free survival than pN stage, especially in patients with high-risk features such as young age, HER2-enriched or triple-negative tumor, and recommended that NR should be preferentially considered in decision making for adjuvant treatment [13].

Although most studies used a value between 0.20 and 0.25 as a minimal cut-off threshold to distinguish risk groups, there is no consensus on which value is the most reliable [5-14]. We used 0.15 as a cut-off value, which may be considered somewhat low. Because the number of positive nodes is inevitably limited in the N1 category, however, the distribution of the NR is strongly affected by the number of nodes sampled. While other studies have focused on patients with between 10 and 16 excised nodes, the present study investigated patients with a median of 18 excised nodes.

Extensive data suggest that other clinico-pathologic findings also can predict an increased risk of locoregional recurrence and even distant metastasis, such as young age, higher histologic grade, negative hormone receptors, presence of ECE, presence of LVI, and inadequate resection margins [15-19]. Recently, Truong et al. reported that T1-T2 breast cancer patients with one to three positive nodes, young age (< 50 years), histologic grade 3, or ER-negative disease had high 10-year locoregional recurrence risks (up to 20%), even after breast-conserving surgery was followed by whole breast radiotherapy [15]. In the current study, those findings were not significant factors for locoregional recurrence or distant metastasis independently but showed selective significance in adjusted analysis combined with the NR.

Regardless of the extent of surgery, substantially high locoregional recurrence rates have been reported in patients with 1-3 positive nodes [15,20-24]. Locoregional recurrence also has been linked to distant metastasis and long-term breast cancer mortality [25-28]. In a meta-analysis of five National Surgical Adjuvant Breast and Bowel Project (NSABP) trials, patients who experienced locoregional recurrence had a considerably lower 5-year DMFS: 51.4% after ipsilateral breast tumor recurrence, 31.5% after axillary recurrence, and 12.1% after supraclavicular metastasis, respectively [27]. Data from the Breast Cancer Trialists' Collaborative Group (BCTCG) showed the overall absolute reduction of 5-year locoregional recurrence by 19%, resulting in a 5% overall absolute reduction of 15-year breast cancer mortality risk in patients who underwent either breast-conserving surgery or mastectomy [28]. In the current study, the HNR group showed lower LRRFS, DMFS, and DFS. However, it is inconclusive whether decreased risk of distant metastasis resulted from decreased locoregional recurrence because only a small number of patients experienced locoregional recurrence.

The National Cancer Institute of Canada Clinical Trials Group (NCIC-CTG) has suggested that adding regional RT may improve survival compared with whole breast RT only when administered after breast-conserving surgery in patients who have T1-T2 breast cancer with N1 or moderate to high risk N0 [29]. The current study revealed that regional RT reduced the risk of distant metastasis in the HNR group only; however, this finding could also support the interpretation that regional RT is unnecessary for LNR patients who have undergone adequate axillary dissection and had no poor prognostic factors. For optimization of the locoregional modality, it is necessary to better define the selection criteria for adjuvant RT. The NR may be a useful indicator for deciding whether to use adjuvant regional RT to treat patients with N1 disease.

Inadequate nodal sampling (less than 10), histology grade 3, estrogen receptor-negative breast carcinomas, or presence of LVI are all considered to be related to the risk of regional recurrence. Previous studies have shown that sampling fewer than 10 axillary nodes is associated with an increased risk of subsequent locoregional recurrence [15,23,24,30]. Tai et al. included in their study only patients with 10 or more excised nodes in order to avoid the possibility of an increased regional relapse rate resulting from understaging or undertreatment [6]. The adjuvant regional RT could compensate for the compromised regional control resulting from inadequate axillary dissection; however, this result does not directly apply to patients in the HNR group who have undergone adequate axillary dissection and remain at substantial risk for locoregional recurrence [31].

## Conclusions

The results of this study associate a NR > 0.15 with increased risk of disease recurrence, especially in young patients with unfavorable pathologic profiles.

## Abbreviations

LN: lymph node; BCS: breast-conserving surgery; MRM: modified radical mastectomy; SLNBx: sentinel lymph node biopsy; ALND: axillary lymph node dissection; CMF: cyclophosphamide/methotrexate/5-fluorouracil; FEC: 5-FU/epirubicin/cyclophosphamide; FAC: 5-FU/adriamycin/cyclophosphamide; ACT: adriamycin/cyclophosphamide/paclitaxel; NR: nodal ratio; IDC: infiltrating ductal carcinoma; ER: estrogen receptor; PR: progesterone receptor; ECE: extracapsular extension; LVI: lymphovascular invasion; RM: resection margin NED: no evidence of disease; LRR: locoregional recurrence; DM: distant metastasis; LRRFS: locoregional recurrence-free survival; DMFS: distant metastasis-free survival; DFS: disease-free survival

## Competing interests

The authors declare that they have no competing interests.

## Authors' contributions

IAK designed this study and is responsible for the preparation of manuscript with TJH. TJH, EYK and WJ contributed to the management of clinical data. SWK, JHK, YJK, JSK, and IAK provided clinical expertise in clinical breast oncology. SYP contributed to the pathologic work. All authors read and approved the content of manuscript.
